# The interconnected roles of TRIM21/Ro52 in systemic lupus erythematosus, primary Sjögren’s syndrome, cancers, and cancer metabolism

**DOI:** 10.1186/s12935-023-03143-x

**Published:** 2023-11-22

**Authors:** Chueh-Hsuan Hsu, Yung-Luen Yu

**Affiliations:** 1 Graduate Institute of Biomedical Sciences, College of Medicine, China Medical University, Taichung, 40402 Taiwan; 2 Institute of Translational Medicine and New Drug Development, Taichung, 40402 Taiwan; 3 Center for Molecular Medicine, China Medical University Hospital, Taichung, 40402 Taiwan; 4 Department of Medical Laboratory Science and Biotechnology, Asia University, Taichung, 41354 Taiwan

**Keywords:** TRIM21/Ro52, Anti-Ro52 antibody, Systemic lupus erythematosus, Primary Sjögren’s syndrome, Cancer immunology, Cancer metabolism

## Abstract

Protein tripartite motif-containing 21 (TRIM21/Ro52), an E3 ubiquitin ligase, is an essential regulator of innate immunity, and its dysregulation is closely associated with the development of autoimmune diseases, predominantly systemic lupus erythematosus (SLE) and primary Sjögren’s syndrome (pSS). TRIM21 /Ro52 also features anti-cancer and carcinogenic functions according to different malignancies. The interconnected role of TRIM21/Ro52 in regulating autoimmunity and cell metabolism in autoimmune diseases and malignancies is implicated. In this review, we summarize current findings on how TRIM21/Ro52 affects inflammation and tumorigenesis, and investigate the relationship between TRIM21/Ro52 expression and the formation of lymphoma and breast cancer in SLE and pSS populations.

## Introduction

TRIM21/Ro52, which is encoded by a gene on the short arm of chromosome 11, is a member of the TRIM protein family, the members of which are involved in multiple cellular functions, including apoptosis signaling, the regulation of innate immunity, and the suppression or activation of carcinogenesis [1, 2]. The structure of TRIM21/Ro52, a cytosolic Fc receptor, consists of an N-terminal RING domain with E3 ubiquitin ligase activity, a B-box domain, a central coiled-coil domain, and a PRY/SPRY domain at its C terminus [3, 4]. TRIM21/Ro52 is crucial in antigen presentation and the regulation of innate immunity in response to intracellular pathogens as a negative regulator of interferon production via the ubiquitination of interferon regulatory factor (IRF)3/5/7, which leads to subsequent proteasomal degradation and prevents further IFN transcription [5–7].

TRIM21/Ro52 and the antibodies that target it (anti-TRIM21/Ro52) are involved in many autoimmune diseases, especially rheumatic diseases, such as systemic lupus erythematosus (SLE) and primary Sjögren’s syndrome (pSS) [8]. The presence of anti-TRIM21/Ro52 is one of the key items to validate the diagnosis of pSS [9], and it can be detected in 42–50% of SLE patients [10]. In addition to the diagnostic role in SLE and pSS, TRIM21/Ro52 can have opposing effects in different cancers [11]. Higher expression of TRIM21/Ro52 is associated with better patient survival in some cancer types, such as diffuse large B-cell lymphoma (DLBCL), breast cancer, and renal cell carcinoma [12–14]. In contrast, TRIM21/Ro52 promotes cancer cell proliferation and migration in glioma and thyroid cancer, and it increases drug resistance in colorectal and pancreatic cancers [15–17].

Heterogeneity is a characteristic shared by SLE, pSS, and cancers at the phenotypic and genotypic levels. Meanwhile, growing evidence indicates that reprogramming cellular metabolism and immune dysfunction both contribute to autoimmune diseases and tumor development [18, 19]. Recent studies have suggested a higher risk of not only hematologic cancers but also solid tumors such as lung cancer and thyroid cancer in individuals with either SLE or pSS. On the other hand, the risk of certain types of malignancies, breast cancer, for example, is decreased compared with healthy controls in other data [20, 21].

**Fig. 1:**
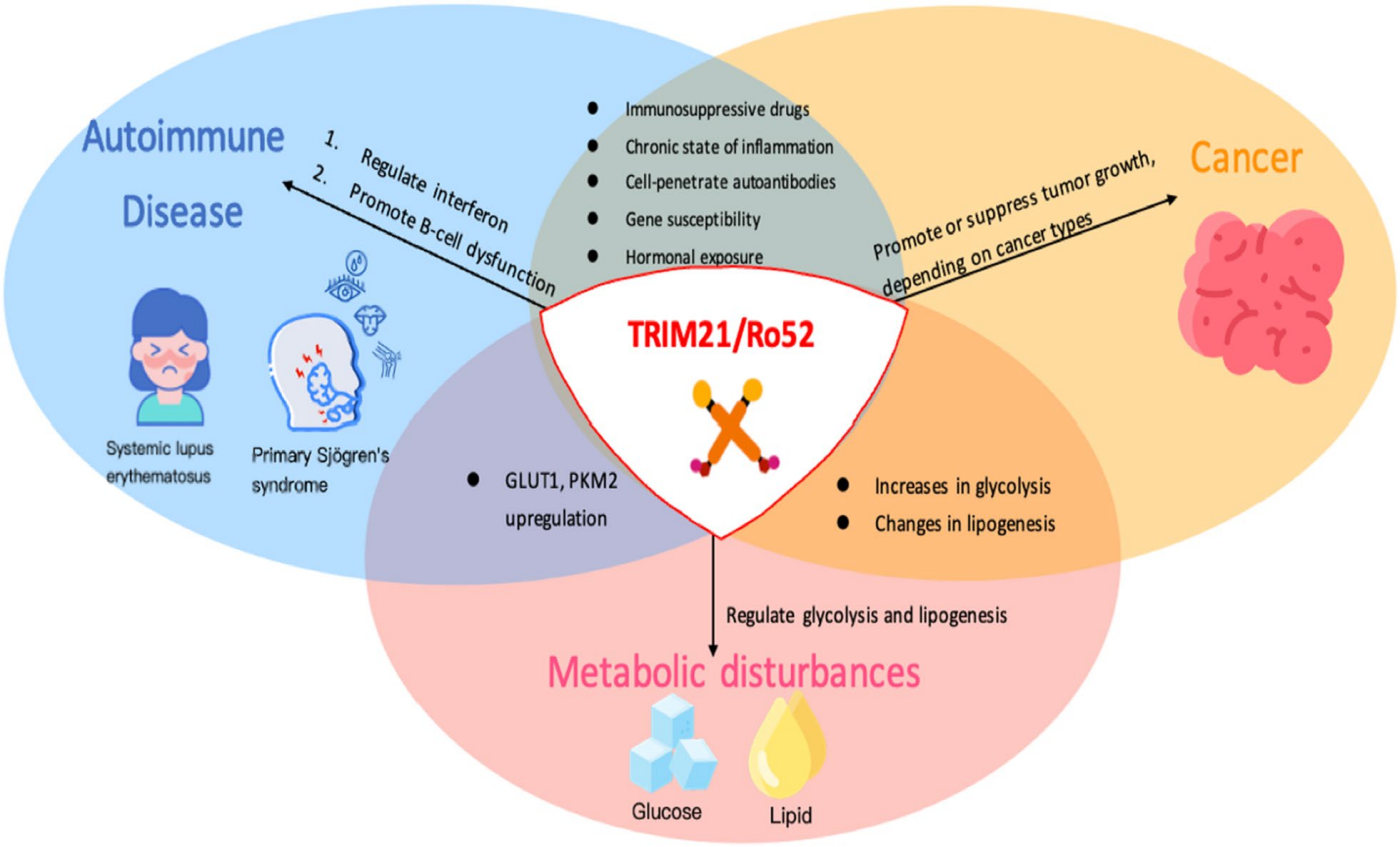
Graphic summary of the interconnected roles of TRIM21/Ro52 in systemic lupus erythematosus, primary Sjögren’s syndrome, cancers, and metabolism

The pathophysiologic mechanisms by which SLE and pSS patients have a greater risk of certain cancers have yet to be well understood, and the comprehension of the regulatory role of TRIM21/Ro52 in these diseases and malignancies remains partial. Herein, in this review, we summarize current evidence of TRIM21/Ro52 relevant connections to immune regulation and cellular metabolism in SLE, pSS, and cancers, then discuss how TRIM21/Ro52 may function in the tumorigenesis, especially lymphoma and breast cancer in SLE and pSS patients (Fig. 1).

## The role of TRIM21/Ro52 in SLE and pSS

### TRIM21/Ro52 expression in individuals and animal models with SLE or pSS

SLE and pSS are both systemic rheumatic diseases characterized by abnormal B-cell activation and autoantibody production. The mRNA level and surface expression of TRIM21/Ro52 protein, which was initially noted in apoptotic cells [22], is higher in freshly isolated peripheral blood mononuclear cells of pSS patients as compared with those of healthy donors [23]. Transcription of TRIM21/Ro52 is upregulated by interferon regulatory factor (IRF)1 and 2 while suppressed by IRF4 and IRF8, which is consistent with the significantly higher level of IRF1 and IRF2 and overexpression of TRIM21/Ro52 seen in patients with Sjögren’s syndrome [24]. In addition, overexpression of TRIM21/Ro52 inhibits cell proliferation and enhances apoptosis in CD40-mediated cell death, which also results in a more significant amount of autoantigen that may trigger a greater autoimmune response [23].

However, deficiency in TRIM21/Ro52 expression is another SLE and pSS development mechanism. Ro52-null (Ro52^–/–^) mice, which are generated from one of the lupus mouse models and detected by green fluorescent protein(GFP) expression, develop progressive dermatitis from the site of their ear tag injury and later are positive for proteinuria detection and deposition of immune complexes as based on renal pathology [25]. The immune response of Ro52^–/–^ mice to a contact-sensitizing agent is also significantly higher than that of Ro52^+/+^ mice, which includes a larger amount of T-cell activation and proinflammatory cytokine production. This TRIM21/Ro52 deficiency increases proinflammatory cytokine production, and the inflammatory infiltration depends on the IL-23–Th17 pathway [26].

### The regulatory role of TRIM21/Ro52 in SLE and pSS

TRIM21/Ro52 plays a regulatory role in B-cell proliferation and differentiation. Ro52−/− mice show increased B-cell activation and markedly higher antibody production than wild-type [27]. Previous studies have reported that TRIM21/Ro52 directly regulates several IRFs that are involved in B-cell development, and the presence of TRIM21/Ro52 could suppress the canonical NF‐κB pathway via monoubiquitinating the phosphorylated IκB kinase subunit beta(IKKβ) and subsequent autophagy [28, 29]. Thus, the absence of TRIM21/Ro52 results in aberrant NF-κB activation, which is also explicitly required for the proliferation of activated B-cell DLBCL, the main subtype of DLBCL found in SLE patients [30–32]. In another study, researchers noted that Ro52^–/–^ mice have enhanced resting B-cell differentiation and exhibit a lupus-like disease phenotype with increased urinary protein and serum double-stranded DNA antibody titers. In the same study, the authors demonstrated that SLE patients who are positive for anti-TRIM21/Ro52 have a significantly higher level of resting B-cell differentiation toward plasmablasts and increased antibody production in general as compared with seronegative SLE patients and healthy controls, indicating that anti-TRIM21/Ro52 impairs the TRIM21/Ro52 function of B-cell homeostasis [33].

Under normal physiologic circumstances, TRIM21/Ro52 negatively regulates type I IFN expression via degradation of IRF3/5; however, the IRF degradation process is downregulated in anti-TRIM21/Ro52 positive SLE patients with both increased TRIM21/Ro52 mRNA and IFN levels, whereas there is no correlation in the SLE population without these antibodies. This suggests that the autoantibody targeting TRIM21/Ro52 impairs TRIM21/Ro52-dependent IRF degradation, resulting in dysregulated IFN production and subsequent high TRIM21/Ro52 expression [34]. A study also showed that the presence and elevated serum level of anti-TRIM21/Ro52 in SLE and pSS patients are positively correlated with the abnormally increased titer of immunoregulatory cytokine, including IL-2, IL-4, IL-21, IL-22, and CXCL10 [35]. The incidence rate and the trigger associated with the production of nuclear-penetrating autoantibodies have not been sufficiently studied yet, whereas the mechanisms by which autoantibodies penetrate cells have been widely investigated and include both Fc receptor–dependent endocytosis and Fc receptor–independent pathways involving myosin and the nucleoside transporter, respectively [36].

### Anti-TRIM21/Ro52 and the link to clinical presentations

Clinically, anti-TRIM21/Ro52 antibodies serve as a potential diagnostic and prognostic biomarker. In pSS patients, higher serum level of anti-TRIM21/Ro52 antibodies are associated with increased disease severity, including a greater rate of anemia and muscular involvement, despite no clear association between the presence of anti-TRIM21/Ro52 antibodies and xerostomia, one of the most notable clinical presentation found on pSS patients [37]. However, it is demonstrated in another study that higher anti-TRIM21/Ro52 antibody titers are actually correlated with severe involvement in parotid scintigraphy, parotid enlargement, and positive salivary gland biopsy, which are all important for pSS diagnostics, indicating a strong association of histopathological findings with anti-TRIM21/Ro52 antibodies [38]. Furthermore, in patients with suspected autoimmune diseases, higher isolated positive serum anti-TRIM21/Ro52 antibodies are associated with a higher diagnostic rate of immunologic disorders and malignancies as compared with the anti-Ro52 and anti-Ro60 double-positive population [39].

## Association of TRIM21/Ro52 and cancers

### TRIM21/Ro52 inhibits tumorigenesis in multiple cancers

TRIM21/Ro52 has a dual role in cancers, as it can promote or suppress tumor growth depending on cancer cell types. The downregulation of TRIM21/Ro52 is associated with a poor prognosis in patients with certain kinds of cancer, including DLBCL, breast cancer, gastric cancer, renal cell carcinoma, ovarian cancer, and colitis-associated cancer [12–14, 40–43] (Table 1).

**Table 1:** The role of TRIM21/Ro52 in different cancers and its effect on clinical outcomes

TRIM21/Ro52 role in different cancers	
Cancer types	Correlation between TRIM21/Ro52 upregulation and clinical outcomes
Glioma [15], Thyroid cancer [16], Pancreatic cancer [17]	Upregulation of TRIM21/Ro52 would enhanced cancer cell proliferation and drug resistance, resulting in poor overall survival
DLBCL [12], Renal cell carcinoma [14, 50], Gastric cancer [41], ovarian cancer [42], breast cancer [44–46]	Upregulation of TRIM21/Ro52 can suppress tumor proliferation and migration, and increase drug sensitivity, leading to better overall survival

In breast cancer, overexpression of TRIM21/Ro52 promotes ubiquitination and degradation of Snail, resulting in the downregulation of E-cadherin transcription throughout the epithelial–mesenchymal transition process, thereby inhibiting migration and invasion capabilities in breast cancer cells [44]. TRIM21/Ro52 also negatively regulates the SET domain containing 7, histone lysine methyltransferase (SETD7, also known as SET7/9), which is involved in breast cancer cell proliferation, migration, and invasion, such that higher expression of TRIM21/Ro52 is associated with better outcomes in these patients [45]. In addition, degradation of spalt-like transcription factor 4 (SALL4), a transcription factor that promotes breast cancer cell proliferation and migration, is regulated by TRIM21/Ro52, and thus higher levels of TRIM21/Ro52 can reduce SALL4 levels [46]. The proteasomal degradation of transforming growth factor beta receptor 2 (TβRII) protein is also mediated by TRIM21/Ro52. By disrupting the TGF-β signaling pathway, TRIM21/Ro52 can suppress triple-negative breast cancer cell metastasis [47]. In addition, lower TRIM21/Ro52 expression in mutant p53 breast cancer patients is associated with a poorer clinical outcome as TRIM21/Ro52 deficiency leads to the accumulation of mutant p53 and the subsequent breast cancer progression [48].

In gastric cancer, higher TRIM21/Ro52 expression not only is correlated with a lower recurrence and a better 5-year survival rate but also enhances the chemosensitivity of the tumor cells to apatinib, an FDA-proved treatment for chemotherapy-refractory advanced gastric cancer [41, 49]. Similarly, colitis-associated and ovarian cancer studies indicate that TRIM21/Ro52 can suppress intestinal epithelial carcinogenesis and ovarian tumorigenesis, respectively [42, 43]. In addition, in renal cell carcinoma, overexpression of TRIM21/Ro52 destabilizes hypoxia-inducible factor 1 subunit alpha (HIF-1α), leading to the suppression of aerobic glycolysis and subsequent inhibition of both in vitro and in vivo tumor cell proliferation and migration [14]. A recent study, in contrast, found that TRIM21/Ro52 inhibits the expression of the lipogenic enzyme via the degradation of sterol regulatory element binding transcription factor 1 (SREBF1), attenuating lipogenesis and tumor growth in renal cell carcinoma [50]. These results also suggest the essential role of TRIM21/Ro52 in cancer metabolism, which will be further discussed in section "The role of TRIM21/Ro52 in cancer cell metabolism".

### TRIM21/Ro52 acts as an oncogene in glioma, thyroid, and pancreatic cancer

In contrast to the findings described above, in the case of certain cancers—such as glioma, thyroid cancer, and pancreatic cancers—overexpression of TRIM21/Ro52 is associated with unfavorable clinical outcomes. In the context of glioma, TRIM21/Ro52 overexpression promotes cell cycling, proliferation, and migration of glioma cells by suppressing the p53–p21 pathway [15]. TRIM21/Ro52 plays a similar role in thyroid cancer, such that the expression of TRIM21/Ro52 is upregulated in thyroid cancer tissue, and higher TRIM21/Ro52 levels are associated with a higher risk of recurrence and lymph node metastasis, though the mechanism involved has not yet been investigated [16]. It was also observed in colorectal and pancreatic tumor cells that TRIM21/Ro52 overexpression in response to cisplatin, a widely used chemotherapeutic agent, would downregulate the level of pro-apoptotic WT1 regulator (PAWR), which is a tumor suppressor mediating apoptosis regulation in various cancer cells, and thus increases the resistance of colorectal and pancreatic tumor cells to cisplatin treatment. It is also demonstrated that high TRIM21/Ro52 expression in pancreatic tumor patients indicates worse survival outcome [17].

### Cancer type with controversial TRIM21/Ro52 function

The function of TRIM21/Ro52 in colorectal cancer (CRC) and hepatocellular carcinoma (HCC) remains contradictory. TRIM21/Ro52 is found to interact with DLGAP1 antisense RNA 2 (DLGAP1-AS2), a long noncoding RNA, and promote the CRC cells' growth and metastasis [51]. In contrast, it is shown in CRC that TRIM21/Ro52 mediates the ubiquitination of MICAL-like 2 protein (MICALL2), which is proven to promote CRC cell proliferation and migration, and thereby the presence of TRIM21/Ro52 decrease the activity of MICALL2 in CRC tumorigenesis [52]. Another study also demonstrates that TRIM21/Ro52 ubiquitinates IGF2 mRNA binding protein 3 (IGF2BP3), which plays an essential role in CRC development, and that the use of the natural drug Berberine improves CRC by promoting the expression of TRIM21/Ro52 [53].

The study by Ding et al. showed that the downregulation of TRIM21/Ro52 contributes to hepatocarcinogenesis and is associated with a poor prognosis [40]. In contrast, Qi et al. found the opposite: the expression of TRIM21/Ro52 is higher in HCC tissues than in normal control tissues, and it is significantly correlated with tumor progression in HCC patients [54]. Furthermore, one target of the E3 ubiquitin ligase activity of TRIM21/Ro52 is p62, and the function of the downstream p62–Keap1–Nrf2 antioxidant pathway in HCC is also controversial; again, the expression of TRIM21/Ro52 has been shown to both involve in promoting and suppressing HCC progression [55, 56]. Further studies are needed to identify the functional role of TRIM21/Ro52 in HCC.

In addition, it is worth noting that TRIM21/Ro52 is an intracellular Fc receptor with extremely high affinity for IgG antibodies, which may lead to the co-precipitation of not just proteins directly interacting with TRIM21 but also the antibodies themselves, leading to a false positive result in co-immunoprecipitation (Co-IP) experiments for identifying potential interaction proteins of TRIM21 [57]. Future studies investigating on the proteins interplay involving TRIM21/Ro52 may require rigorous experimental design and validation steps using alternative methods such as bioluminescence resonance energy transfer (BRET) or a mutated PRY/SPRY domain of TRIM21/Ro52 with reduced affinity for IgG to rule out non-specific binding [58].

### The role of anti-TRIM21/Ro52 antibodies in cancers

There have been conflicting results regarding the function of anti-TRIM21/Ro52 antibodies in different cancers [59, 60]. Anti-TRIM21/Ro52 positive is associated with poor survival in patients with esophageal squamous cell carcinoma [60]. In another study, although there was a significantly high prevalence of anti-TRIM21/Ro52 in ovarian cancer patients, the presence of this antibody was correlated with higher overall survival compared with the antibody-negative ovarian cancer population [61]. As increased TRIM21/Ro52 expression was associated with better outcomes in ovarian cancer in recent research, whether the antibody impairs TRIM21/Ro52 protein function or the presence of anti-TRIM21/Ro52 antibodies reflects the overexpression phenomenon of TRIM21/Ro52 in cancer requires further investigation [42].

## Cancer profile and its pathophysiological mechanisms in SLE and pSS

### Cancer epidemiology study and mechanisms of tumor development in SLE and pSS individuals

The overall cancer risk in SLE and pSS patients is slightly higher than that of their matched general population. Among all cancer types, hematologic cancers, especially non-Hodgkin lymphoma, are associated with the most significant increase in risk [62, 63]. SLE and pSS patients have a fourfold and an up to 44-fold higher risk of developing B-cell lymphoma, respectively [64, 65]. Concerning solid neoplasms, SLE patients have a higher incidence rate of lung cancer, cervical cancer, and cervical dysplasia. In contrast, the risk of mouth and throat cancer, thyroid cancer, and lung cancer is notably increased in patients with pSS [66, 67]. In contrast, the risk of hormonal-related cancers, which include breast, prostate, endometrial, and ovarian cancers, is lower in SLE and pSS patients than in their healthy counterparts [67, 68] (Table 2). This characteristic may be attributed to the shorter period of hormonal exposure in these patients due to premature ovarian insufficiency [69]; undiscovered genetic variants; and the use of nonsteroidal anti-inflammatory drugs (NSAIDs), which have a protective effect against breast cancer [70], in SLE and pSS patients [71]. However, there is still a lack of solid evidence, and the breast cancer risk in SLE and pSS patients shows striking geographical differences [72, 73].

**Table 2:** Cancer risk profiles in SLE and pSS patients

	Systemic lupus erythematosus	Primary Sjogren’s syndrome
Increased risk	All hematologic cancers (Including lymphoma, leukemia, multiple myeloma), Lung cancer, Liver and hepatobiliary cancer, Vaginal and vulvar cancer, Cervical dysplasia and precancerous lesions, Thyroid cancer, Head and neck cancer, Renal cancer, Nonmelanoma skin cancer	All hematologic cancers (Including lymphoma, leukemia, multiple myeloma), lung cancer, Thyroid cancer Lip and oropharyngeal cancer, Liver cancer, Nonmelanoma skin cancer
Inconsistent findings	Bladder cancer, Cervical cancer, Breast cancer, Ovarian cancer	Breast cancer, Bladder cancer, Ovarian cancer,
Decreased risk	Prostate cancer, Endometrial cancer, Melanoma	

Pathophysiological mechanisms underlying the increased risk of non-hormonal-related malignancies, particularly hematologic cancers, in SLE or pSS patients may involve the use of certain immunosuppressive drugs, chronic inflammatory status, susceptibility of genes, cell-penetrating autoantibodies, and other conventional shared risk factors such as smoking and Epstein-Barr virus infection [74–76]. It has been confirmed that a dose-dependent use of cyclophosphamide increased the risk of hematologic malignancies in SLE patients. In contrast, hydroxychloroquine protects patients with SLE, and systemic glucocorticoid, cyclophosphamide, methotrexate, or azathioprine was not correlated with an increased cancer risk [77–79]. In pSS patients, there is limited study on the question of cancer risk and the use of immunosuppressive agents, but it has been reported that hydroxychloroquine presents a neutral effect on cancer development in the pSS population [80]. In addition, immunosuppressive treatments may indirectly promote oncogenic virus infections [81].

The pathogenesis of SLE involves the overexpression of a myriad of cytokines and the subsequent dysregulation of B-cell proliferation and differentiation, which overlaps with the formation of DLBCL, the most common type of lymphoma found in SLE patients [31, 82]. The production of free radicals due to neutrophil activation and the promotion of proinflammatory cytokines in SLE and pSS patients also lead to direct DNA damage, which may contribute to certain malignancies [83, 84]. On the other hand, it has been established that in pSS patients not only T and B lymphocytes, but the salivary epithelial cells aberrantly produce B cell-activating factor (BAFF) which leads to B cell hyperactivity and clonal expansion of B cells [85, 86]. Overactivation of B cells results in a wide spectrum of autoantibodies, such as rheumatoid factor, an antibody against the Fc portion of IgG, or autoantibodies against other autoantigens including TRIM21/Ro52 and SSA/Ro60, and the accumulation of these immune complexes may in return give rise to chronic antigenic stimulation and activation of NF‐κB pathway [87, 88]. As previously mentioned, TRIM21/Ro52, which is aberrantly expressed in SLE and pSS patients, regulates B cell homeostasis though NF‐κB pathway and thus drives the formation of lymphoma, especially mucosa-associated lymphoid tissue (MALT) lymphomas and DLBCL [89, 90].

The genomes of patients with SLE and pSS are more likely to have polymorphisms at specific loci that encode DNA repair factors and cytokine regulators, and these polymorphisms not only contribute to the development of autoimmune diseases but also may increase the risk of cancer [91, 92]. The gene that encodes A20, or TNF alpha-induced protein 3 (TNFAIP3), an immunoregulatory factor involved in the downregulation of the NF-κB pathway and the carcinogenesis of lymphoma and solid tumors, often includes a single-nucleotide polymorphism and one gene mutation in SLE patients that are absent in healthy individuals [93]. Furthermore, autoantibodies that penetrate cells in individuals with autoimmune diseases, may interfere with the tumorigenesis [94, 95]. It has been reported that 3E10, a cell-penetrating lupus anti-DNA antibody, is toxic to BRCA2-deficient cancer cells [94]. On the contrary, antiphospholipid antibodies, which bind to mitochondria through internalization and trigger cell death, are found in 30–40% of SLE patients and associated with an increased risk of hematologic cancers [95, 96].

In addition to the fact that SLE patients are associated with a greater risk of metabolic syndrome, which is a combination of well-acknowledged risk factors for cancer, including hypertension, diabetes, and obesity, changes in immunometabolism in these patients could act as another predisposing factor for developing cancer [97, 98]. Expression of glucose transporter 1 (GLUT1), a critical regulatory component in glucose metabolism, in immune cells is higher in SLE and pSS patients than in healthy controls [97, 99, 100]. The upregulation of GLUT1 is correlated with autoimmune disease severity, and various cancer studies have found it in common [101, 102]. Pyruvate kinase M2 (PKM2), an isoform of the pivotal regulatory enzyme, pyruvate kinase, in cell metabolism, is significantly elevated in the monocytes, dendritic cells, and B cells of SLE patients relative to that in the general population [103]. Activation of PKM2 is not only involved in toll-like receptors mediated inflammation and autoimmunity but also contributes to cancer formation [103, 104]. The growing amount of study regarding metabolic syndrome in individuals with SLE and pSS has shed light on the pathogenesis of the diseases themselves and provided new avenues for exploring the relationship between cancers and autoimmune diseases.

### The expression of TRIM21/Ro52 and its association with lymphoma and breast cancer development in SLE and pSS patients

**Fig. 2:**
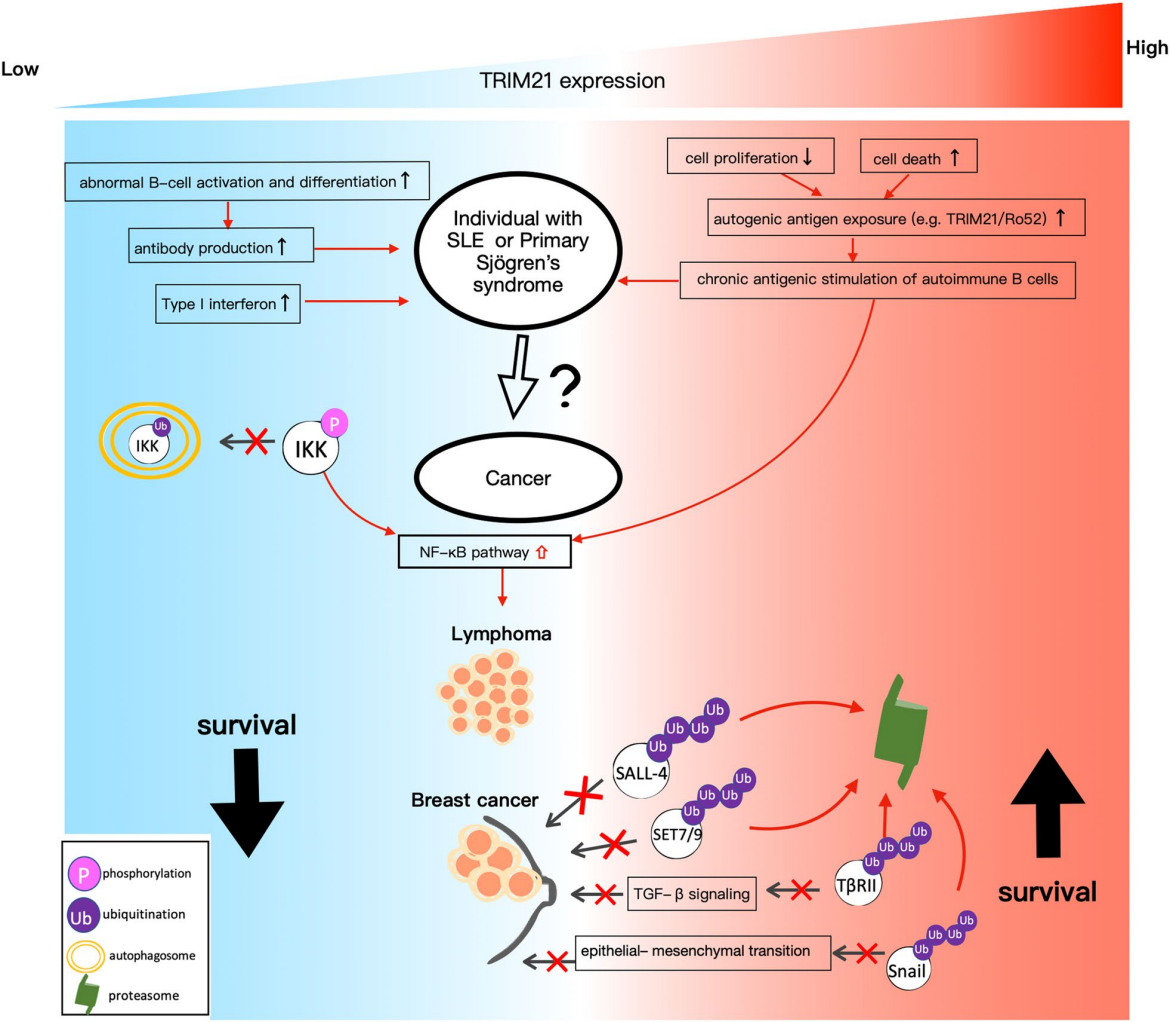
A summarized mechanism(s) that SLE or pSS may lead to an increased risk of lymphoma and a decreased risk of breast cancer. Down-regulation of TRIM21/Ro52 expression is associated with poor overall survival in both lymphoma and breast cancer. IKKβ: kappa‐B kinase subunit beta; SALL-4: spalt like transcription factor 4; SET7/9: SET domain containing 7, histone lysine methyltransferase; TβRII: growth factor beta receptor 2

In SLE and pSS patients, the risk of developing lymphoma is significantly elevated. Ro52–/– mice, which serve as a model for SLE, have demonstrated both lupus-like symptoms and aberrant B-cell differentiation and proliferation [33]. TRIM21/Ro52 deficiency may be relevant to the increased risk of lymphoma because of NF‐κB pathway activation, and maintaining TRIM21 expression is also associated with a preferable clinical outcome in patients with lymphoma [12, 29]. It is, however, paradoxical that the expression of TRIM21 mRNA and protein in SLE and pSS populations is found to be higher than that in healthy controls, and this increased level of TRIM21 could result in increased cell death and enhanced autogenic antigen exposure with subsequent antigen stimulation of autoimmune B cells, thus serving as a connection with the development of lymphoma [105] (Fig. 2). These results suggest that lymphomagenesis in individuals with SLE and pSS is a multistep and multifactorial process and that maintaining the balanced expression of TRIM21 is essential for both the development of autoimmune diseases and the formation of lymphoma.

The relationship between SLE and breast cancer represents an interesting contrast based on regional differences. Research from some Asian countries, such as Taiwan and Korea, demonstrates an increased risk of breast cancer in SLE patients [72, 106]. In contrast, other multi-center cohort studies, primarily conducted in the US and European countries, show decreased breast cancer risk among the SLE population [71]. Since it is demonstrated that there is higher TRIM21 expression among individuals with SLE or pSS and that several studies confirmed that overexpression of TRIM21 is associated with the inhibition of breast cancer development and a better prognosis for those individuals who develop breast cancer, it is worth further investigation of the TRIM21 expression level in SLE and pSS patients in these Asian countries.

## The role of TRIM21/Ro52 in cancer cell metabolism

Cancer cells often exhibit a higher rate of glycolysis than normal cells to meet the energetic needs associated with rapid growth. This metabolic change is known as the Warburg effect [107]. In human glioblastoma, blocking of TRIM21/Ro52 activity due to protein kinase B (AKT) activation impairs the proteasomal degradation of phosphofructokinase-1 (PFK1), the rate-limiting glycolysis enzyme, resulting in the promotion of glycolysis and brain tumor proliferation [108]. Regardless of the changes in the cellular microenvironment, there is a tendency for TRIM21/Ro52 inactivation to result in higher PFK1 expression and higher glycolysis rates in human non-small-cell-lung-cancer cells [109].

TRIM21/Ro52 is not only essential for the regulation of glucose metabolism but also has a pivotal role in lipogenesis. Fatty acid synthase (FASN), a complex of critical enzymatic proteins in the fatty acid synthesis pathway, is commonly upregulated in tumor cells to support the need for lipids for active proliferation, and increased FASN expression is correlated with drug resistance, tumor metastasis, and reduced survival among individuals with cancer. TRIM21/Ro52 is responsible for the polyubiquitination and proteasome degradation of FASN [110]. A study by Gu et al., which examined the underlying mechanism of how FASN promotes lipogenesis and HCC progression, found that the acetylation of glyceronephosphate *O*-acyltransferase (GNPAT), a critical enzyme that regulates plasmalogens, stabilizes FASN via suppressing both TRIM21/Ro52-mediated GNPAT and FASN degradation [111].

The upregulation of the pentose phosphate pathway is another metabolic change that frequently occurs in cancer cells in response to the high level of reactive oxygen species [112]. TRIM21/Ro52 is responsible for the degradation of glucose-6-phosphate dehydrogenase (G6PD), the rate-limiting enzyme in the pentose phosphate pathway, and its feedback regulation on phosphatidylinositol 3-kinase (PI3K)/AKT pathway makes TRIM21/Ro52 a potential therapeutic target in PI3K/AKT activation cancer [113].

## Conclusion

TRIM21/Ro52 is a commonly seen autoantigen in many systemic autoimmune diseases, especially SLE and pSS patients. Though the mechanisms of autoimmune disease development and cancer formation remain elusive, two significant characteristics shared by both diseases are chronic inflammatory status and metabolic dysfunction. An emerging role of TRIM21/Ro52 in the regulation of inflammation and the reprogramming of cellular metabolism has been indicated in previous studies.

The underlying mechanism of how TRIM21/Ro52 may lead to cancer development or provide a protective effect against cancer in SLE and pSS patients requires further studies. Identifying the interconnected role of TRIM21/Ro52 between SLE, pSS, and tumorigenesis would be beneficial, as this could help develop an appropriate cancer monitoring method for patients with autoimmune diseases and may shed light on the disease development of SLE and pSS themselves.

## Data Availability

Not applicable.

## Abbreviations

BAFF: B cell-activating factor
CRC: Colorectal cancer
DLBCL: Diffuse large B-cell lymphoma
DLGAP1-AS2: DLGAP1 antisense RNA 2
FASN: Fatty acid synthase
GFP: Green fluorescent protein
GLUT1: Glucose transporter 1
G6PD: Glucose-6-phosphate dehydrogenase
GNPAT: Glyceronephosphate *O*-acyltransferase
TβRII: Growth factor beta receptor 2
HCC: Hepatocellular carcinoma
HIF-1α: Hypoxia-inducible factor 1 subunit alpha
IGF2BP3: IGF2 mRNA binding protein 3
IRF: Interferon regulatory factor
IKKβ: Kappa‐B kinase subunit beta
MICALL2: MICAL like two protein
MALT: Mucosa-associated lymphoid tissue
NSAIDs: Nonsteroidal anti-inflammatory drugs
NF-κB: Nuclear factor kappa-light-chain-enhancer of activated B cells
PI3K: Phosphatidylinositol 3-kinase
PFK1: Phosphofructokinase-1
PSS: Primary Sjögren’s syndrome
PAWR: Pro-apoptotic WT1 regulator
AKT: Protein kinase B
PKM2: Pyruvate kinase M2
SET7/9: SET domain containing 7, histone lysine methyltransferase
SALL-4: Spalt-like transcription factor 4
SREBF1: Sterol regulatory element binding transcription factor 1
SLE: Systemic lupus erythematosus
TNFAIP3: TNF alpha-induced protein 3
